# Genome-Wide Discovery of InDel Markers in Sesame (*Sesamum indicum* L.) Using ddRADSeq

**DOI:** 10.3390/plants9101262

**Published:** 2020-09-24

**Authors:** Sibel Kizil, Merve Basak, Birgul Guden, Hilal Sule Tosun, Bulent Uzun, Engin Yol

**Affiliations:** 1 Department of Field Crops, Faculty of Agriculture, Akdeniz University, TR-07058 Antalya, Turkey; sibl.kzl@gmail.com (S.K.); basak_1129@hotmail.com (M.B.); birgulguden@akdeniz.edu.tr (B.G.); bulentuzun@akdeniz.edu.tr (B.U.); 2 Department of Plant Protection, Faculty of Agriculture, Akdeniz University, TR-07058 Antalya, Turkey; hilaltosun@akdeniz.edu.tr

**Keywords:** genetic diversity, insertion, deletion, marker, oil crop, sesame

## Abstract

The development and validation of different types of molecular markers is crucial to conducting marker-assisted sesame breeding. Insertion-deletion (InDel) markers are highly polymorphic and suitable for low-cost gel-based genotyping. From this perspective, this study aimed to discover and develop InDel markers through bioinformatic analysis of double digest restriction site-associated DNA sequencing (ddRADSeq) data from 95 accessions belonging to the Mediterranean sesame core collection. Bioinformatic analysis indicated the presence of 7477 InDel positions genome wide. Deletions accounted for 61% of the InDels and short deletions (1–2 bp) were the most abundant type (94.9%). On average, InDels of at least 2 bp in length had a frequency of 2.99 InDels/Mb. The 86 InDel sites having length ≥8 bp were detected in genome-wide analysis. These regions can be used for the development of InDel markers considering low-cost genotyping with agarose gels. In order to validate these InDels, a total of 38 InDel regions were selected and primers were successfully amplified. About 13% of these InDels were in the coding sequences (CDSs) and in the 3′- and 5′- untranslated regions (UTRs). Furthermore, the efficiencies of these 16 InDel markers were assessed on 32 sesame accessions. The polymorphic information content (PIC) of these 16 markers ranged from 0.06 to 0.62 (average: 0.33). These results demonstrated the success of InDel identification and marker development for sesame with the use of ddRADSeq data. These agarose-resolvable InDel markers are expected to be useful for sesame breeders.

## 1. Introduction

Sesame (*Sesamum indicum* L.) is an oilseed plant in the family Pedaliaceae that has been cultivated for thousands of years. Sesame has been called “the queen of oil seeds” [1] because of its high levels of nutritional oils and proteins [2]. The oil content of most genotypes ranges from 35% to 60% [3], and the highest reported value is 62.7% [4]. Sesame seed oil has five major fatty acids: oleic acid (C18:1), linoleic acid (C18:2), stearic acid (C18:0), palmitic acid (C16:0), and arachidic acid (C20:0) [5]. Oleic acid and linoleic acids are the predominant fatty acids [6]. In addition, sesame oil contains several unique antioxidant lignans (sesamin and sesamolin), which may reduce the risk of atherosclerosis, cardiovascular disease, and coronary heart disease [7]. These polyphenols also provide resistance to oxidative deterioration [8] and are therefore highly important for the oil industry. Sesame plants grow well in tropical and subtropical climates, can tolerate low soil moisture, require low labor input, and can be grown in pure or mixed stands with diverse crops [9]. Despite these advantages, sesame yield is very low due to the persistence of wild-type traits—nonsynchronous flowering, capsule shattering in harvest [10], susceptibility to phyllody disease [11], indeterminate growth [12], late maturation, and low environmental adaptability [13,14]. The paucity of genetic diversity in sesame species, in addition to the limited amount of basic research, breeding studies, and international cooperation, have hindered efforts to improve agronomically important traits in sesame species.

DNA markers are highly reliable tools that can provide more rapid and accurate characterization of plants than traditional methods [15]. Researchers have used DNA markers in studies of genomic mapping, biodiversity, and gene tagging. Although sesame is an economically important crop, its improvement by use of DNA markers has lagged behind other major oil crops because it is mainly grown in developing countries [16]. Useful molecular markers, including random amplified polymorphic DNA (RAPD) [17], amplified fragment length polymorphism (AFLP) [18,19], simple sequence repeat (SSR) [16,20,21,22], sequence-related amplified polymorphism (SRAP) [23], and insertion-deletion (InDel) [24,25] markers, have been developed and widely used in genetic diversity studies. A few mapping and marker-assisted selection studies have also been conducted with the use of AFLP markers for the closed capsule mutant trait [26]; RAPD markers for corolla color [27]; and SSR markers for determinate growth habit [12], male-sterile gene [28], and oil and protein content [2] to improve the efficiency of sesame breeding programs. A large number of single nucleotide polymorphisms (SNPs) have also been identified with the advent of next-generation sequencing technology and have been used for the exploitation of genetic diversity [29,30], the construction of high-density linkage mapping [31,32,33], and the identification of candidate genes for the improvement of sesame production [34,35,36,37].

Corresponding regions of genes and genomes in different plants can have different sequence lengths because of insertions or deletions [38]. These mutations are called InDels, and can be formed by insertion of transposable elements, unequal crossover events between similar repeat copies, or slippage in simple sequence replication [39], and may manifest as loss of function or a non-sense mutation [40]. InDels and SNPs are the most abundant and widely distributed sources of variability in plant genomes [41]. They are highly suitable for mapping, genome-wide association analysis, and other genetic studies. However, InDels are preferable to SNPs in marker-assisted breeding programs because InDel polymorphisms can be visualized with more readily designed primers, basic PCR systems, and agarose gel electrophoresis [42]. There is also evidence of greater polymorphism of InDel markers than SSR markers in sesame [25]. Although previous researchers have used InDel markers in studies of many different crops [43,44,45], only a few studies have examined the use of these markers in sesame [24,25,46]. Consequently, we attempted to develop InDel markers with the use of double digest restriction site-associated DNA sequencing (ddRADSeq) data from 95 sesame accessions compared with a reference genome sequence. The selected markers were also validated on sesame germplasm to evaluate their efficiency.

## 2. Results

We performed quality filtering and then generated 349.86 M raw sequence reads by sequencing 95 sesame accessions using the Illumina HiSeq platform. Among these accessions, the mean number of reads was 3.68 M and the guanine-cytosine (GC) content was 38% [30]. We processed these filtered data using bioinformatic analysis and identified 7477 InDel sites (Table 1).

Deletions accounted for 61% of these InDel positions. Their sizes ranged from 1 to 14 bp, and 94.9% had sizes of 1 to 2 bp. Single-nucleotide variation was the most common type, followed by bi-nucleotide insertions, and these two types accounted for more than 93% of the total insertions. Among all InDels, 97.5% were less than 5 bp, 2.2% were between 5 to 10 bp, and 0.4% were more than 10 bp long. Single nucleotide length InDels may arise from read or alignment errors, therefore we separately assessed the statistics for each InDel of which the length was greater than a single nucleotide (Table 2).

The frequency of InDels that were at least 2 bp long varied among the chromosomes, with the greatest number in chromosome 3 and the smallest number in chromosome 13 (Table 2). Separate analysis of insertions and deletions of this size indicated that chromosome 3 also had the greatest numbers of deletions and insertions, and chromosome 13 had the smallest numbers of deletions and insertions. The frequency of InDels of this size varied among chromosomes, and ranged from 2.19 InDels/Mb (chromosome 13) to 4.10 InDels/Mb (chromosome 3). We examined InDels of 8 bp and longer for development of InDel markers, based on consideration of their genomic distribution and low-cost genotyping with agarose gels (Table 3).

There were 86 InDel sites with a length ≥8 bp found in the sesame genome (Table 3) and more than half of these were deletions. Chromosome 11 had the highest number of deletions (9) and chromosome 3 had the most insertions (6). We detected no ≥8-bp deletions in chromosome 10 and no ≥8-bp insertions in chromosome 5 (Table 3). The chromosomal position, sequence, and size information for insertions and deletions are shown in Table 4 and Table 5. The longest insertion (13 bp) was in chromosome 9 (physical position: 4042652) and the longest deletions (14 bp) were in chromosome 1 (physical position: 12141886), chromosome 5 (physical position: 12064933), and chromosome 11 (physical positions: 9853924 and 11733409). Identified InDels of length ≥8 bp were analyzed in Integrated Genome Browser (IGB) software to display the regions in their appropriate genomic positions (Figures S1 and S2).

A total of 38 InDel regions with lengths ≥ 8 bp were selected and primers were successfully designed with the primer3 package. These 38 InDels were distributed throughout the 13 chromosomes. Chromosomes 1 and 8 had the greatest number of InDel markers (6), followed by chromosome 9 (5). The clean amplicons were generated with these primers (Figure S3). Analysis of InDel genomic positions indicated that 86.84% of them were in intergenic regions, 7.90% were in 5′ untranslated regions (UTRs), 2.63% were in 3′ UTRs, and 2.63% were in coding sequences (CDS) (Table 6).

**Figure 1:**
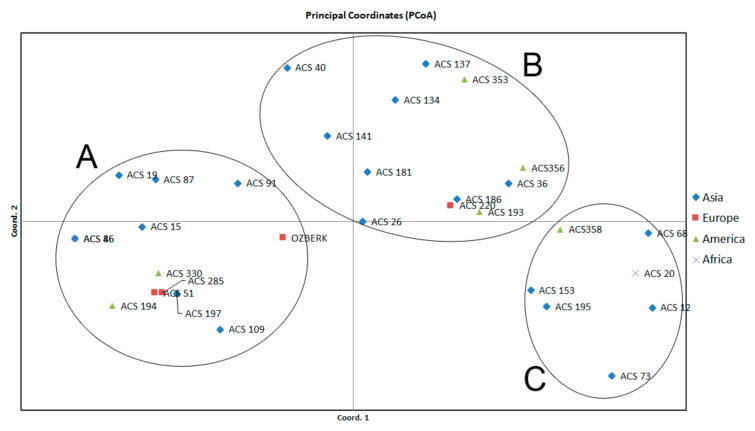
Principal coordinate analysis (PCoA) of the 32 sesame accessions genotyped with 16 InDel markers. (**A**–**C**) show the three classes with respect to distribution of genotypes.

**Figure 2:**
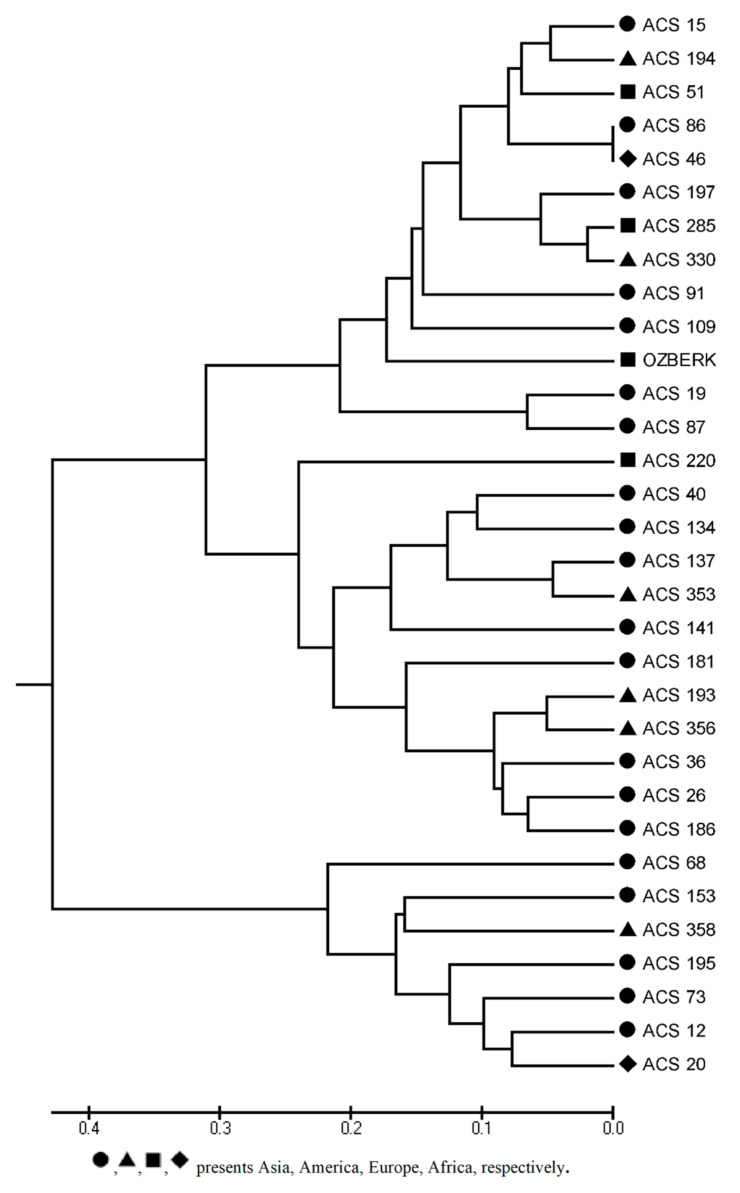
UPGMA dendrogram showing the genetic relationships among the 32 sesame accessions.

A genetic diversity analysis was conducted with 16 InDel markers across 32 randomly selected sesame accessions from the Mediterranean sesame core collection. PCR products were visualized on a Fragment Analyzer^®^ for all the studied loci (Figure S4) and InDel markers showed the expected polymorphisms within the accessions (Table 7). The observed and expected heterozygosities were found in the range of 0 to 0.25 and 0.02 to 0.47, respectively. The highest expected heterozygosity value was obtained in loci S-D-5-157, S-D-7-143, and S-D-8-223, and the lowest was seen in loci S-D-1-106 and S-I-4-120, with a mean value of 0.34. The average Shannon diversity index (I) was found to be 0.49. The polymorphic information content (PIC) values of these 16 markers ranged from 0.06 to 0.62, with an average of 0.33. Principal coordinate analysis (PCoA) indicated that the first and second coordinate explained 27.66% and 14.93% of the total variation, respectively. The sesame panel was also divided into three groups including accessions from different continents in the PCoA graphic (Figure 1). The UPGMA tree also showed two distinct groups (Figure 2).

## 3. Discussion

ddRADSeq is a cost-effective sequencing protocol that uses two restriction enzymes to reduce genome complexity for SNP discovery and genotyping [47]. We used ddRADSeq to identify 7477 InDel sites, with the ddRADSeq indicating the effectiveness of this protocol to identify InDel regions in the sesame genome. To our knowledge, this is the first successful large-scale development of InDel markers in sesame using ddRADSeq data. The InDels we identified varied among chromosomes, confirming the suitability of this protocol for genome-wide marker development. Therefore, they can be used for the construction of high-density genetic maps, the exploitation of genetic diversity, and the identification of candidate genes. We also presented an optimized procedure for InDel detection using the Galaxy platform (www.usegalaxy.org) that does not require coding processes with stringent bioinformatics settings.

Table 1 showed that a total of 14 InDel classes were detected based on type (insertion vs. deletion) and the number of InDels declined with the increase of InDel size, and the most common type were single-nucleotide InDels. These results are in concordance with previous studies which reported that single-nucleotide InDels were most common in kenaf [48], chickpea [49], and sesame [24]. In contrast, bi-nucleotide InDels were most common in *Zea mays* [50] and *Brassica rapa* [44]. Our analysis of InDels that were at least 2 bp long indicated the greatest number in chromosome 3 and the smallest number in chromosome 13. This observation is consistent with previous studies of sesame, which reported that the greatest number of simple sequence repeats (SSRs) [16] and SNPs [30] were on chromosome 3. On the other hand, we identified no deletions that were 8 bp or longer in chromosome 10 and no deletions of this length in chromosome 5. This might be a disadvantage of ddRADSeq, because there can be large gaps in the genome coverage after sequencing of a genomic library prepared using this protocol [51]. Our InDel frequency was 1 per 37.74 kb (7477 InDels in 259.73 Mb), much higher than the frequency (1/137 kb) obtained by Wei et al. [24], who used transcriptome assembly for InDel detection in sesame. In addition, we found more InDels compared to a study which used restriction site-associated DNA (RAD) sequencing [25]. These differences, therefore, could be a consequence of the sequencing method, the number of genotypes used for genotyping, and bioinformatic parameters for the exploration of variants.

Plant breeders commonly accept agarose gel-based DNA markers more than those markers from newer technologies, such as HRM, KASP, SNP arrays, and PAGE-based SSR, due to the ease of use and the familiarity of the agarose gel system [52]. This led us to develop 38 agarose-resolvable markers and successful amplifications were obtained with bulk DNAs. The lack of PCR failure in individual PCR assays indicates the absence of variation in primer binding sites. In turn, this further shows the power of the ddRADSeq library approach and the InDel filtering pipeline, leading to 100% success in PCR assays. In addition, we used a single PCR program for amplifying multiple loci, suggesting the potential utility of these markers for multiplex PCR assays. Annotation analysis showed that most of the InDels were in intergenic regions (Table 6), similar to the results of Wei et al. [24], who developed InDel markers from sesame transcriptome data. About 13% of the developed InDels were in the CDS and the 3′ and 5′ UTRs (Table 6), suggesting that they may be valuable resources for genomics-assisted breeding applications. For example, researchers previously reported an 11-bp deletion in the early flowering 3 gene (*ELF3*) of chickpea and successfully used this region as an InDel marker [53].

The exploitation of genetic diversity in sesame genetic resources is highly important in order to utilize collections and improve breeding studies. In this study, the effectiveness of the developed markers was assessed on the sesame germplasm, including 32 accessions from four different continents. Genetic diversity analysis showed that the average PIC value of 16 markers was 0.33, higher than PIC value of the InDel (Wei et al. [54]) and AFLP (Laurentin and Karlovsky [55]) markers used to identify genetic variation in sesame. Previous research also reported a PIC value above 0.50 for SSR markers [56,57] and expressed sequence tag-SSR (EST-SSR) markers [20] in sesame. Botstein et al. [58] categorized the PIC values of markers as highly informative (≥0.5), reasonably informative (0.50 to 0.25), or least informative (≤0.25). Our average PIC value (0.33) thus indicates that the markers identified here are reasonably informative and adequate for evaluating relationships among accessions, according to Meszaros et al. [59]. The principal coordinate analysis using 16 InDel markers between 32 sesame accessions revealed three classes, giving no clear pattern with respect to geographical origin. Migration of different accessions by people and/or trade among regions over centuries may explain these results [30]. Previous research also reported that human-related factors may be responsible for the lack of correlation between genetic and geographical distance in other crop plants [60]. Our findings are in agreement with the conclusions of Laurentin and Karlovsky [18], who reported no association between genetic differentiation and accession origin in sesame. Most of the sesame accessions used in PCoA and UPGMA tree analysis based on genetic distance from 16 InDel markers were consistent with a phylogenetic tree analysis conducted with 5292 SNPs [30]. This demonstrates the effectiveness of the new markers, which successfully revealed differences among accessions in the present investigation. In addition, InDel markers showed their ability to reliably discern genetic diversity in sesame collections [25].

## 4. Materials and Methods

### 4.1. Plant Material and DNA Extraction

The Mediterranean sesame core collection consists of 103 accessions, and previous studies have characterized their agro-morphological traits [61], oil characteristics [62], and SNP data [30]. The core collection was developed with the principal component score strategy from 345 sesame accessions, considering 12 qualitative and nine quantitative traits [61]. The seeds of each accession in the collection were sown in pots; however, eight of them did not germinate. The remaining 95 accessions in the collection, which were from 21 different regions in Africa, America, Asia, and Europe, were used as a genetic material for ddRADSeq analysis (Table S1). DNA was extracted from young leaves using the CTAB method [63] with minor modifications. The quality and quantity of DNA was checked by electrophoresis on 1% agarose gels, and the amount was normalized to 100 ng/μL using lambda DNA as a reference.

### 4.2. ddRADSeq and InDel Calling

Before genotyping, random DNA samples were tested with *MspI* to determine the effectiveness of restriction enzyme digestion. A ddRAD library was prepared using restriction enzymes (*Vsp*I and *Msp*I) using a modification of the ddRAD method [47], as described by Basak et al. [30]. A reduced representative genomic library with an insert size of 400–500 bp was subjected to Illumina 150-bp paired-end sequencing. The ddRAD sequencing data of 95 available genotypes (accession number: PRJNA560319) were submitted to the National Center for Biotechnology Information (NCBI) Sequence-Read Archive (SRA) database.

For bioinformatic analysis, the raw data were demultiplexed using Je V1.2 [64], a quality check was conducted for FASTQ Sanger files using fastp [65], and reads with a Phred quality score less than 15 out of 40 and restriction enzyme sequences were trimmed. Each genotype was subsequently aligned with the reference genome sequence “Zhongzhi13 V2.0” [66] using Bowtie2 with default parameters [67] in the Galaxy software framework (www.usegalaxy.org). The resulting BAM files were used in Freebayes (Galaxy Version 1.1.0.46–0) [68], with simple diploid calling and filtering, and coverage values of 20× for variant calling. The resulting variant files were filtered using VCFfilter (Galaxy Version 1.0.0) and SNPs were discarded. The individual .vcf files, which included insertions and deletions, were later merged using VCFgenotypes (Galaxy Version 1.0.0) to form a single data file.

The combined variant file was processed using Microsoft Excel to eliminate duplicated regions and organize the InDels according to their sizes. InDel regions that were at least 8 bp long were checked using the Integrated Genome Browser V9.1.4 (IGB) [69] with each BAM file and the sesame reference genome.

### 4.3. Primer Design and PCR Analysis

Forward and reverse primers from sesame reference genome sequences that flanked the selected InDels were designed using Primer3Plus ([70]; http://www.bioinformatics.nl/cgi-bin/primer3plus/primer3plus.cgi) to develop genome-wide InDel markers. The length of primer pairs was limited to a minimum of 18 bp and the predicted products ranged from 100 to 900 bp. The primer pairs were later controlled for possible duplication of sequences in the genome using IGB software. All markers were named using the format S-D(I)-X-XXX, where “S” indicates sesame, “D” and “I” indicate deletion and insertion, “X” is the chromosome number, and “XXX” is start of the chromosomal position. InDel annotation was based on the sesame reference genome sequence using Generic Feature Format 3 (GFF3) [66].

Primers were checked with random bulk DNAs, and PCR was performed in a 20 μL reaction volume with 1 μL of 10× PCR buffer, 2.5 mM MgCl_2_, 0.3 μL of dNTP mix (10 mM), 0.3 μL each of forward and reverse primers (10 μM), 0.2 μL of Taq DNA polymerase (5 U/μL), and 1 μL of genomic DNA and Milli-Q water. Thermocycling started at 95 °C for 2.5 min; followed by 4 cycles of 95 °C for 45 s, 50 °C for 20 s, and 60 °C for 50 s; 30 cycles of 92 °C for 20 s, 50 °C for 20 s, and 60 °C for 50 s; and a final extension at 60 °C for 10 min. The PCR products were separated on 2% agarose gels and visualized by UV light.

To determine their performance, 16 selected InDel markers were used to examine the genetic diversity of the sesame germplasm (32 accessions). PCR conditions were performed as described above. The expected PCR bands were monitored using a Fragment Analyzer^TM^ (Advanced Analytical Technologies GmbH, Heidelberg, Germany) for accurate sizing. The DNF-900-K0500 reagent kit was used for qualitative analysis of DNA fragments. The solutions, buffers, and gels were prepared according to the manufacturers’ instructions. The data were normalized to 1 bp (lowest) and 500 bp (highest), and calibrated to the 1 to 500 bp range DNA ladder. The virtual gel image was assessed using PROSize 2.0 (Advanced Analytical Technologies, AMES, IA, USA).

### 4.4. Genetic Diversity Analysis

Calculations of population genetic parameters, number of alleles (Na), effective number of alleles (Ne), Shannon diversity index (I), expected heterozygosity (He), observed heterozygosity (Ho), Wright’s fixation index (F), and principal coordinate analysis (PCoA) were performed using GenAlex V6.5 [71]. The Excel Microsatellite Toolkit [72] was used to measure polymorphism. A phylogenetic tree was constructed based on genetic distance with MEGA 5 [73].

## 5. Conclusions

In this study, a large number of InDels were detected from sequencing of the Mediterranean sesame collection with the use of a ddRADSeq protocol. These results indicated that this technique is an effective approach for the development of genome-wide markers in a short time. Among 86 InDel sites that had lengths of ≥8 bp, 38 agarose-resolvable markers were successfully amplified and 16 of them were randomly selected to detect polymorphism among 32 sesame accessions. The remaining InDel genomic regions (Table 4 and Table 5) identified in this study can therefore be used for the development of InDel markers that might play an important role in different breeding studies, such as the construction of linkage maps, marker-assisted selection (MAS), and gene mapping and selection.

## Figures and Tables

**Table 1:** The number of insertions-deletions (InDels) identified with double digest restriction site-associated DNA sequencing (ddRADSeq) analysis of 95 sesame accessions.

InDel Type	Indel Size (bp)	Number	Frequency (%)
Insertion	1	2573	88.12
	2	143	4.90
	3	77	2.64
	4	44	1.51
	5	24	0.82
	6	13	0.45
	7	13	0.45
	8	6	0.21
	9	9	0.31
	10	6	0.21
	11	5	0.17
	12	6	0.21
	13	1	0.03
	Total	2920	
Deletion	1	4119	90.39
	2	204	4.48
	3	72	1.58
	4	56	1.23
	5	33	0.72
	6	10	0.22
	7	10	0.22
	8	9	0.20
	9	17	0.37
	10	12	0.26
	11	5	0.11
	12	4	0.09
	13	2	0.04
	14	4	0.09
	Total	4557	

**Table 2:** Distribution of insertions-deletions (size ≥2 bp) in the genome.

Chromosome	Number of InDels	Number of Deletions	Number of Insertions	Frequency (InDels/Mb)
Chr1	75	41	34	3.70
Chr2	54	32	22	2.93
Chr3	106	61	45	4.10
Chr4	52	29	23	2.53
Chr5	40	23	17	2.41
Chr6	63	32	31	2.43
Chr7	54	30	24	3.22
Chr8	81	51	30	3.09
Chr9	72	40	32	3.15
Chr10	52	27	25	2.67
Chr11	39	20	19	2.77
Chr12	61	31	30	3.74
Chr13	36	21	15	2.19
Total	785	438	347	38.93

**Table 3:** Distribution of insertions-deletions of length ≥8 bp in the genome.

Chromosome	Number of Deletions	Number of Insertions
Chr1	5	5
Chr2	4	1
Chr3	2	6
Chr4	3	2
Chr5	3	0
Chr6	2	1
Chr7	3	2
Chr8	8	2
Chr9	4	5
Chr10	0	2
Chr11	9	1
Chr12	5	3
Chr13	5	3
Total	53	33

**Table 4:** Information about insertions of length ≥8 bp identified in this study.

Chromosome	Physical Position	Sequence	Size (bp)
Chr1	1924302	AAAAAACAGA	10
Chr1	8602437	TAGTTGAGTAA	11
Chr1	10171409	CTTTTGTTTGC	11
Chr1	15365977	ATAACCCT	8
Chr1	15931209	AAGCATCTGC	10
Chr2	8434594	TCACTTGCTC	10
Chr3	3933997	AAAGATCAT	9
Chr3	5681885	ATAACTTT	8
Chr3	5758231	AATTGTCTG	9
Chr3	13078175	TGGATTGAT	9
Chr3	24847064	CTATCTTGTCTG	12
Chr3	25255054	GTCAGGCG	8
Chr4	3501848	AACAGCAAG	9
Chr4	12047194	TCATAACAATAA	12
Chr6	25170199	TTAGGATATA	10
Chr7	2567633	CGAGTTTAG	9
Chr7	11218635	CGCGCCATGG	10
Chr8	17465130	GTAGGTAATGGC	12
Chr8	22375397	ATGCAGGTATT	11
Chr9	83648	TCCATTCTG	9
Chr9	2878824	TCCCAATTTCG	11
Chr9	4042652	GATCCAGACCTGA	13
Chr9	7344455	AACCTAACTTA	11
Chr9	17977272	ATCTGATTACGT	12
Chr10	1129764	ATTGTTTTACTA	12
Chr10	16879947	CAATTGACA	9
Chr11	12599287	GTTATTACGTGT	12
Chr12	7851788	AAATCCATG	9
Chr12	12737569	AAATCTGT	8
Chr12	14130703	TCTGGGAC	8
Chr13	14413322	TTATTTTCTC	10
Chr13	14462216	TGACTAGA	8
Chr13	14465088	CCTGCTTCT	9

**Table 5:** Information about deletions of length ≥8 bp identified in this study.

Chromosome	Physical Position	Sequence	Size (bp)
Chr1	12141886	ATACATAAATATAT	14
Chr1	10684379	GCGGTCATA	9
Chr1	12499067	TCATATGG	8
Chr1	18076193	TTCAACGCA	9
Chr1	19878537	ATTTTTTATG	10
Chr2	11956920	CACTTAAAT	9
Chr2	16991276	ATCCACGTG	9
Chr2	18254320	GAGTGAGGTTG	11
Chr2	13530605	CTATTCTAGA	10
Chr3	3159274	TTCTTCAGC	9
Chr3	16817209	CCGGTTTTGG	10
Chr4	805760	TTTTCGGCCC	10
Chr4	10090797	CACGAAAGTGAA	12
Chr4	16553051	GTCACCTTTACTG	13
Chr5	4151098	GAAGATGCAT	10
Chr5	12064933	TATATGTCCAAGAA	14
Chr5	15770216	AACCTGAA	8
Chr6	3765637	ACTTGAGT	8
Chr6	15493394	GTTCTTGGGTT	11
Chr7	9663535	TACAGTGA	8
Chr7	13849040	AGGAGGAAT	9
Chr7	14376520	ATTCAGGGC	9
Chr8	204960	AATTATTCTGA	11
Chr8	2232178	GTATGATTAGG	11
Chr8	12091726	CAATGGCTA	9
Chr8	12863799	AATAACACATAA	12
Chr8	17895254	ACCCAAACT	9
Chr8	23088746	CGTATGTAAA	10
Chr8	24437543	CAAAAGCTG	9
Chr8	25510231	ATATTGCC	8
Chr9	1286207	AGGCTTAAC	9
Chr9	17162035	GATGGGTGAG	10
Chr9	22192324	AATCCACAT	9
Chr9	22693423	CCGATTCCGTCA	12
Chr11	1650384	TGCATCCCA	9
Chr11	2176613	GTGATAAGTG	10
Chr11	8335212	CAGGTTCG	8
Chr11	9853924	AATCATACGATGAG	14
Chr11	11435807	GTGCAGAGTA	10
Chr11	13066884	GACCCTGA	8
Chr11	13584022	TTATCAAAT	9
Chr11	13826811	TAAATTTCA	9
Chr12	7621962	TGCACTAAAT	10
Chr12	11308275	AAGAAATTT	9
Chr12	12221515	GCACGACT	8
Chr12	12487265	AGACTAAC	8
Chr12	12936814	AATAACTTAG	10
Chr13	8231260	ACGTCTTGTAGG	12
Chr13	10333430	AATTATTGATC	11
Chr13	10529779	TAACAAGCAGTAA	13
Chr13	12075963	CACCATCAC	9
Chr13	13896532	TGTATCATAA	10

**Table 6:** The primer sequences of the 38 InDel markers developed and used in this study.

Marker Name	Chromosome	Physical Position	InDel Type	Indel Size (bp)	Forward Primer (5′ to 3′)	Reverse Primer (5′ to 3′)	Product Length (bp)	Locus Location *
S-D-1-106	chr1	10684379	Deletion	9	GATGAATTTAATTGAGTCCAACAA	ATTTTTCTGACTTAGGTGTTTATGC	180	UTR_3
S-D-1-121	chr1	12141886	Deletion	14	TTCCAGGTGGAGATCCTGAC	GGAGCGGAATTCTGGACATA	202	intergenic region
S-D-1-124	chr1	12499067	Deletion	8	TTGACGAATAATTTTTGTTTTCCA	CCTGGTGGAAATGGAGTCAA	183	intergenic region
S-D-1-198	chr1	19878537	Deletion	10	TGTGCATCTTTGATACATATGAATTTT	TCACACTGCGTTATTGATTTAATTT	182	intergenic region
S-D-2-135	chr2	13530605	Deletion	10	CAAATTCACATAACCAGCATTGA	GTCCGGGACGTGAAATTGAT	244	intergenic region
S-D-4-805	chr4	805760	Deletion	10	AGGCAGACCAGGGTTTTACA	GGTTTTAGCTCTAGAGGAAAGAAAACT	169	intergenic region
S-D-4-100	chr4	10090797	Deletion	12	GAGCAGCAGCACCCATTAAC	GCAGTGGCTCAATTCTGGTT	231	intergenic region
S-D-4-165	chr4	16553051	Deletion	13	GGGGAAATGATGGAGGGTTA	CAAGTTCAACGTCACCAATTT	249	intergenic region
S-D-5-157	chr5	15770216	Deletion	8	GCGAAACACAGCCTAAAAGG	TGTTTGGAGCTTCCTCATTTG	155	intergenic region
S-D-6-154	chr6	15493394	Deletion	11	GTGTGGCCGGAAATCAAT	TGAAAGCAAACCTCAAGAGTG	234	UTR_5
S-D-7-138	chr7	13849040	Deletion	9	TTTTACCTGGGGATTTGAAGG	CTAACGAGGTGGTGGGCAAT	150	CDS
S-D-7-143	chr7	14376520	Deletion	9	GGATTTAATCGGGGAAGCAT	TCCGATGTTTTCCTTTCGAG	217	intergenic region
S-D-8-223	chr8	2232178	Deletion	11	TCCTACGGTTGGATGTTGATG	ACGGGTGCGCTAACAACC	150	intergenic region
S-D-8-120	chr8	12091726	Deletion	9	CAGGCACCTCAAAGGAAGAG	GGGAGGAGTCGTCTGTCGT	810	intergenic region
S-D-8-178	chr8	17895254	Deletion	9	GTGTGCCCCTAGTTTCGAGT	GTGAGCTGGCGGTGATTATT	198	UTR_5
S-D-8-230	chr8	23088746	Deletion	10	AATTGTATTCGAATCAGGTTTGG	CAGCCATATAGTTGGGTGGA	150	intergenic region
S-D-8-244	chr8	24437543	Deletion	9	TGATTTTGGGATCTTGAACGA	TTGCCTGCTTTATGTGATGC	153	intergenic region
S-D-8-255	chr8	25510231	Deletion	8	TCAAGCCTTAATCGGAGACC	TTCTGCTCTCACGCGTATTC	431	intergenic region
S-D-9-128	chr9	1286207	Deletion	9	TGCATAGCAACATAAATGAGGAA	CTCTTATGCATGGCCACCAC	103	intergenic region
S-D-9-171	chr9	17162035	Deletion	10	CGGAACTTCTCAGTGATAAAGAGC	TCCACCTGTTCCATCCTCTC	353	intergenic region
S-D-12-113	chr12	11308275	Deletion	9	AATTAGCCGCCTTTTTGGTT	TTGTTTTGAAATTGACGGTACG	374	intergenic region
S-D-12-124	chr12	12487265	Deletion	8	TGCATGCATCTAAACCTTGAA	AATTTCGGCACATTTCAAAAA	162	intergenic region
S-D-13-823	chr13	8231260	Deletion	12	GCTTCTTATTCACTTAAATGGTGCT	TCGTCACTTTTTCTAAGAGAGCTT	233	intergenic region
S-D-13-103	chr13	10333430	Deletion	11	TCTCCGGACTGTCTGAAAGG	TGTCTTTGATCCGTTGGTCA	626	intergenic region
S-I-1-101	chr1	10171409	Insertion	11	GGGGAGGTAATTATTCCGTGA	TATACACGTCCGCAAGAGCA	152	intergenic region
S-I-1-192	chr1	1924302	Insertion	10	TCTTCATCTGTCACCCCAAA	CTGTTAAGCGCCACTGTTGA	173	intergenic region
S-I-3-248	chr3	24847064	Insertion	12	TTTTCACCTGTTTCGAGACCT	CTTTGAGCTGGAACGTGGAT	174	intergenic region
S-I-4-120	chr4	12047194	Insertion	12	TTGTTGGAAGGACTAAAATTGAAA	GGGCAATGTGCACCTTTTA	304	intergenic region
S-I-6-251	chr6	25170199	Insertion	10	ATTGCATTTGGGCTGGATTA	CCCCCTCGAAACAACTAATG	228	intergenic region
S-I-7-112	chr7	11218635	Insertion	10	GTCACCCTCAAGGAGATCCA	AAACAGAAAGAAGAGAAAAACCCTTA	238	intergenic region
S-I-8-174	chr8	17465130	Insertion	12	CTGCAAGCAACAAACCAAAA	TCTTCAAGAGCTCATGGCTACA	167	intergenic region
S-I-9-179	chr9	17977272	Insertion	12	CATTCCCTTCAAAACCCACA	TGCAACGCTTGCAAGAAAC	213	UTR_5
S-I-9-404	chr9	4042652	Insertion	13	CAGCGGATTTGTGCTTGTTA	GACTCTAACTTTACCCAATTCTTTAGG	161	intergenic region
S-I-9-836	chr9	83648	Insertion	9	ATGGGCCTGTACCGGTATACTA	TTTTTGAGTGAATGACTATGATTACAT	223	intergenic region
S-I-10-168	chr10	16879947	Insertion	9	TCTATTCTGACATTGACCGGATT	TCACAAAAACAACCAAAGTTGC	152	intergenic region
S-I-10-112	chr10	1129764	Insertion	12	TGATGGAGTAATTGAAAGTGTACG	CAAAAGCAGAGTTGACCGTATG	155	intergenic region
S-I-10-125	chr11	12599287	Insertion	12	GGCAAAGAAATGCAGAGGAG	CACTTTCACCCACCCATCAT	210	intergenic region

* Coding DNA sequence (CDS), untranslated region (UTR).

**Table 7:** Summary of genetic diversity statistics for 32 sesame accessions.

Marker/Locus	Na *	Ne	*I*	Ho	He	F	PIC
S-D-1-106	1.25	1.03	0.05	0.00	0.02	1.00	0.06
S-D-1-121	1.75	1.55	0.45	0.00	0.31	1.00	0.32
S-D-4-165	1.75	1.55	0.45	0.00	0.31	1.00	0.32
S-D-5-157	2.00	1.90	0.66	0.03	0.47	0.95	0.37
S-D-7-143	2.00	1.89	0.66	0.03	0.47	0.94	0.35
S-D-8-223	2.00	1.83	0.64	0.15	0.45	0.61	0.37
S-D-8-178	2.00	1.65	0.54	0.07	0.37	0.64	0.30
S-D-9-128	1.75	1.58	0.47	0.05	0.32	0.84	0.62
S-D-12-124	2.00	1.69	0.58	0.25	0.40	0.33	0.31
S-I-1-192	2.00	1.72	0.59	0.10	0.41	0.66	0.36
S-I-3-248	1.75	1.70	0.51	0.01	0.36	0.96	0.37
S-I-4-120	1.25	1.12	0.13	0.01	0.08	0.82	0.19
S-I-6-251	2.00	1.84	0.63	0.10	0.44	0.67	0.37
S-I-9-179	1.75	1.44	0.40	0.09	0.26	0.51	0.25
S-I-10-168	2.00	1.73	0.60	0.12	0.41	0.71	0.32
S-I-10-112	1.75	1.55	0.46	0.01	0.31	0.96	0.33
Mean	1.81	1.61	0.49	0.06	0.34	0.76	0.33

* Number of alleles (Na), effective number of alleles (Ne), Shannon diversity index (I), expected heterozygosity (He), observed heterozygosity (Ho), Wright’s fixation index (F), polymorphic information content (PIC).

## Supplementary Materials

The following are available online at https://www.mdpi.com/2223-7747/9/10/1262/s1, Figure S1: The software Integrated Genome Browser (IGB) shows the deletion in chromosome 1 (position 10.684.379–10.684.387). Gray colors are deleted sequences for each individual in that region. Coordinates indicate the reference genome sequence. Figure S2: The software IGB shows the insertion in chromosome 10 (position 16,879,947–16,879,956). Green-black colors are inserted sequences for each individual in that region. Coordinates indicate the reference genome sequence. Figure S3: Amplification of sesame DNAs with use of selected markers (Ladder 100 bp), Figure S4: Fragment Analyzer^TM^ shows the sample gel pictures of InDel marker profile for selected sesame accessions with a 1–500 bp ladder. Table S1: List of the sesame accessions in the Mediterranean sesame core collection using ddRADSeq analysis.
