# Supplementary material for: Genome-Wide Discovery of InDel Markers in Sesame (Sesamum indicum L.) Using ddRADSeq
Source: Plants (Basel). 2020 Sep 24;9(10):1262. doi: 10.3390/plants9101262 (PMC7599716; doi:10.3390/plants9101262)
Supplement: Supplementary file 1 [file plants-09-01262-s001.pdf]

plants

MDPI

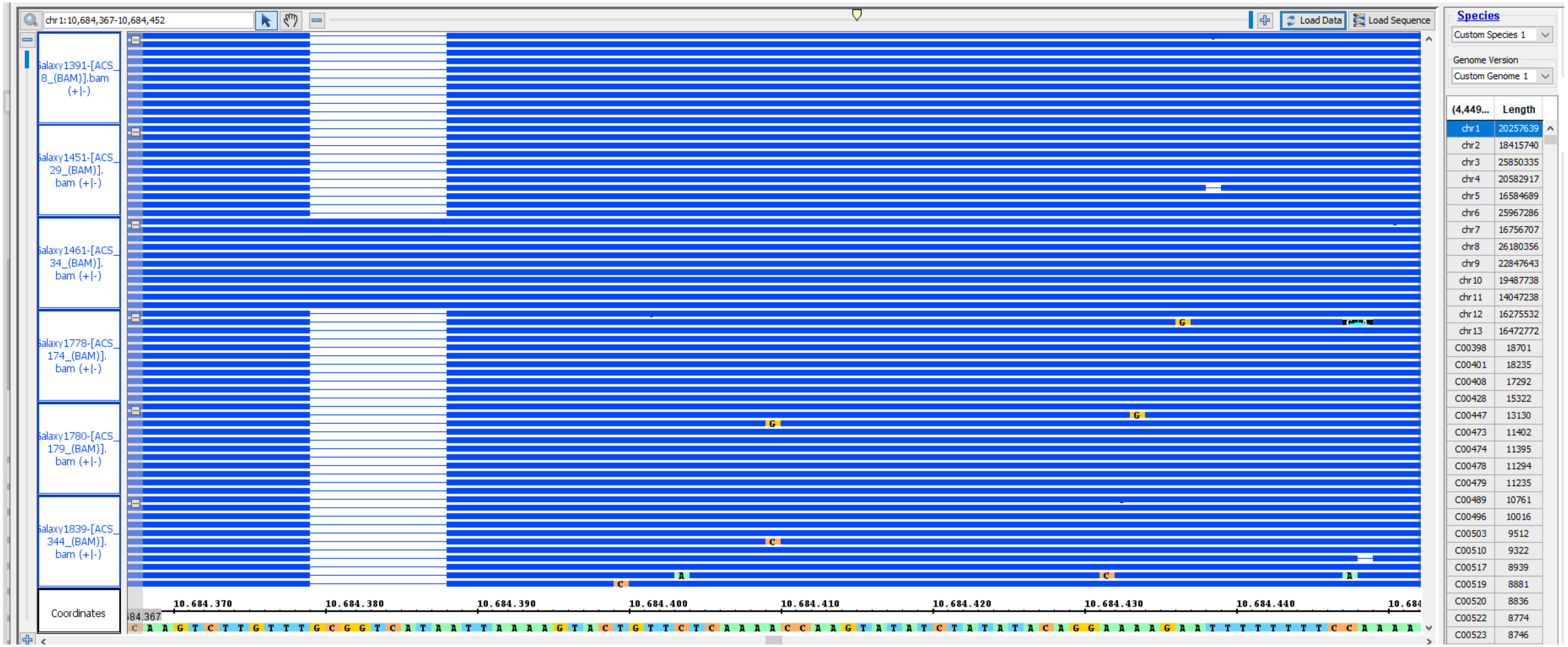

Figure S1. The software IGB shows the representative deletion in chromosome 1 (position 10.684.379-10.684.387). Gray colors are deleted sequences for each individual in that region. Coordinates indicates the reference genome sequence.

plants

MDPI

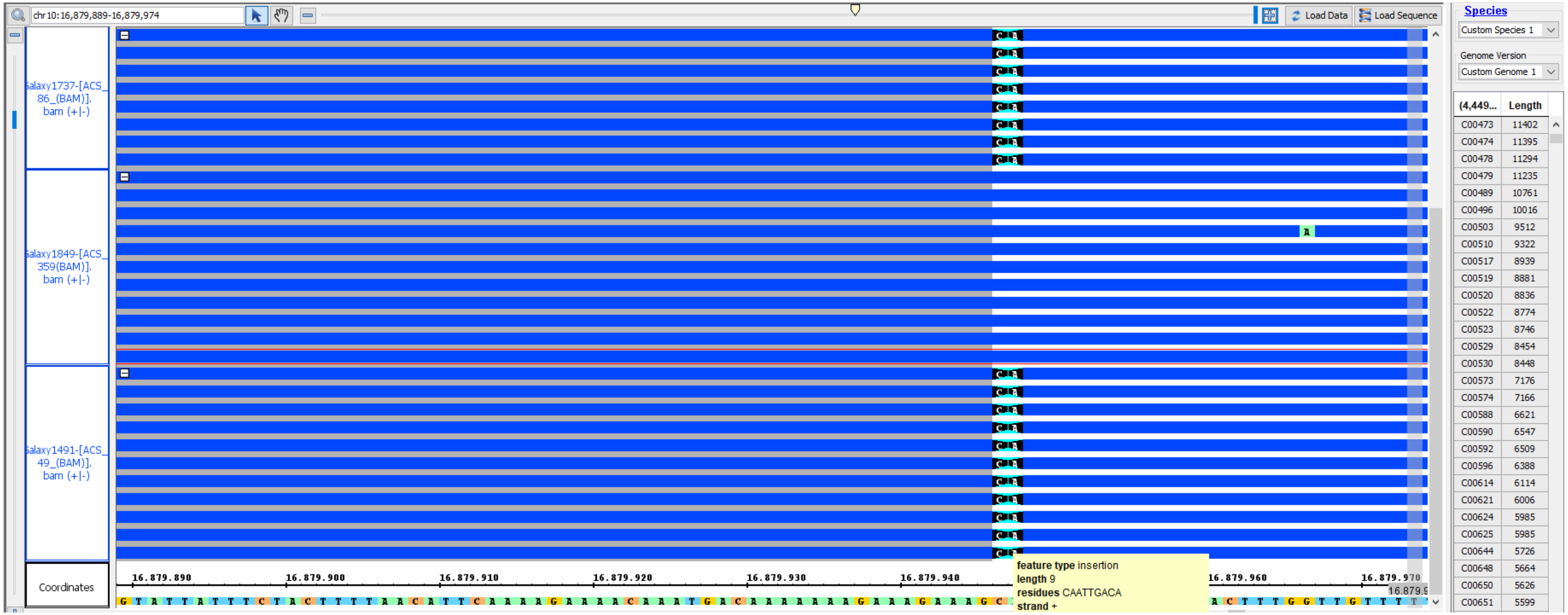

Figure S2. The software IGB shows the insertion in chromosome 10 (position 16,879,947-16,879,956). Green-black colors are inserted sequences for each individual in that region. Coordinates indicates the reference genome sequence.

plants

MDPI

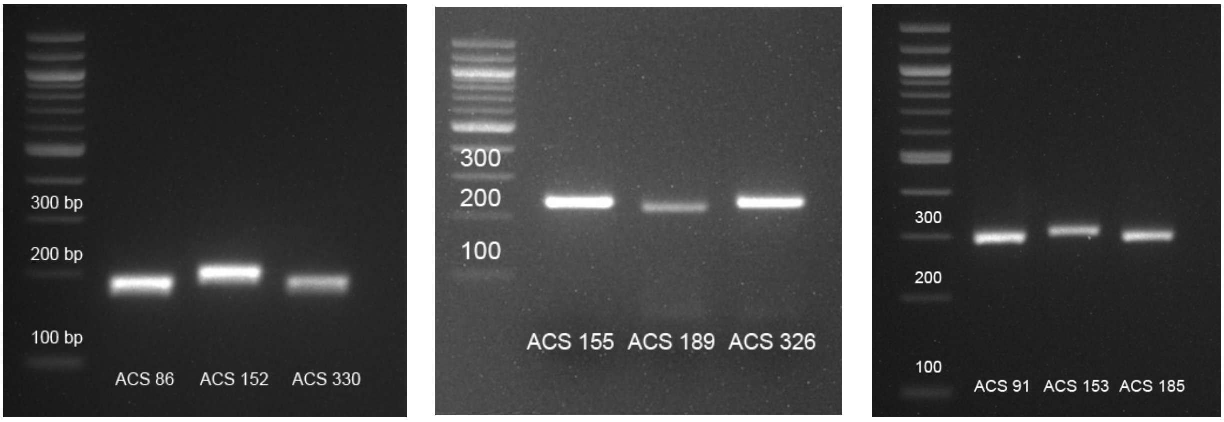

S-D-1-121 S-D-4-100 S-I-4-120

Figure S3. Amplification of sesame DNAs with use of selected markers (Ladder 100 bp)

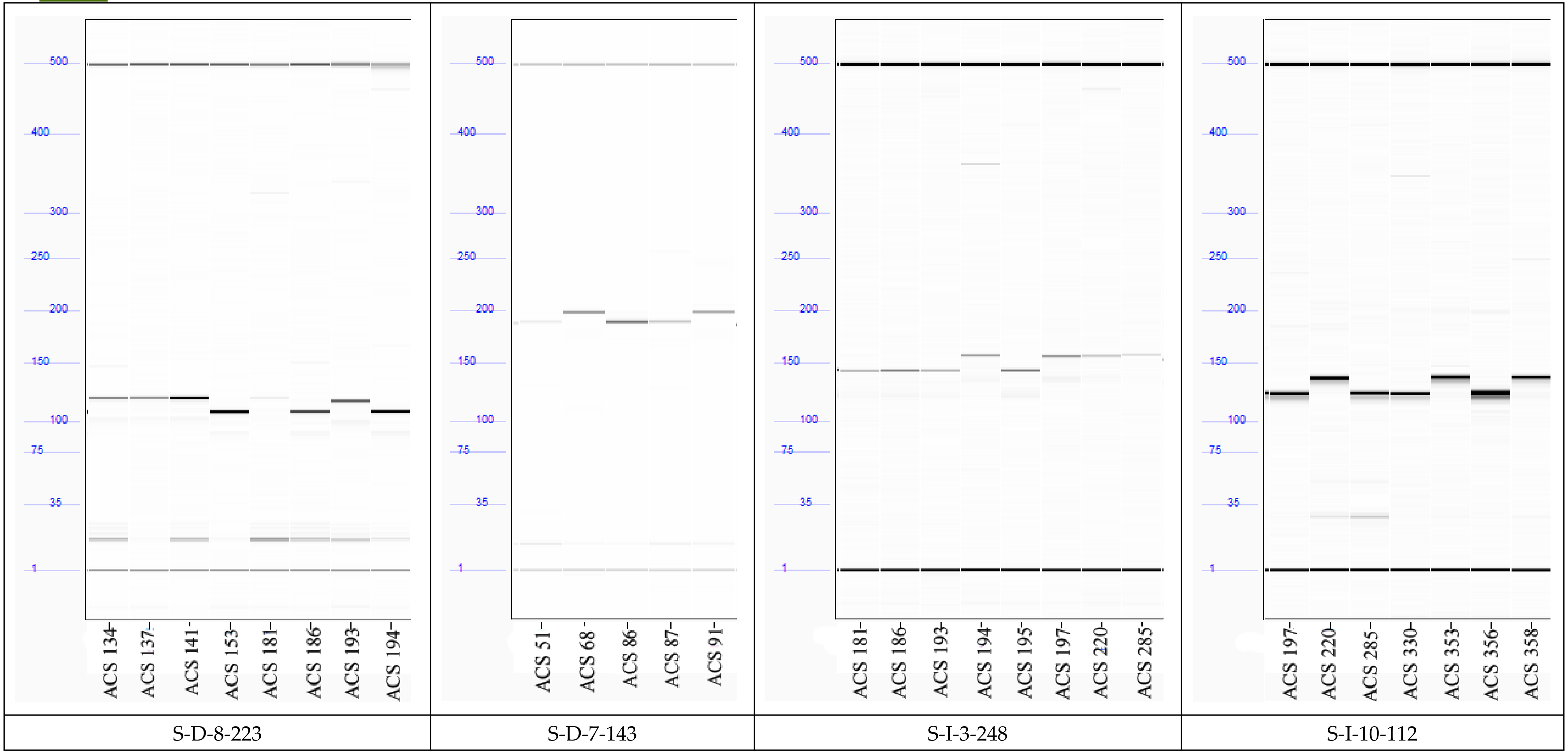

Figure S4. Fragment Analyzer™ shows the sample gel pictures of InDel marker profile for selected sesame accessions with a 1-500 bp ladder.

Table S1. List of the sesame accessions in the Mediterranean sesame core collection used ddRADSeq analysis.

Accession No.	USDA Entry / Cultivar Name	Country of origin	Continent	Accession No.	USDA Entry / Cultivar Name	Country of origin	Continent
ACS 8	PI 220403	Afghanistan	Asia	ACS 157	PI 292147	Pakistan	Asia
ACS 10	PI 220544	Afghanistan	Asia	ACS 158	PI 292148	Pakistan	Asia
ACS 12	PI 223532	Afghanistan	Asia	ACS 159	PI 292149	Pakistan	Asia
ACS 15	PI 223817	Afghanistan	Asia	ACS 163	PI 426941	Pakistan	Asia
ACS 18	PI 426218	Afghanistan	Asia	ACS 164	PI 426942	Pakistan	Asia
ACS 19	PI 426219	Afghanistan	Asia	ACS 168	PI 426967	Pakistan	Asia
ACS 20	PI 278160	Angola	Africa	ACS 174	PI 258369	Russia	Asia
ACS 21	PI 278161	Angola	Africa	ACS 179	PI 263461	Russia	Asia
ACS 22	PI 254710	Argentina	America	ACS 181	PI 263461	Russia	Asia
ACS 24	PI 158045	China	Asia	ACS 182	PI 263465	Russia	Asia
ACS 26	PI 158065	China	Asia	ACS 183	PI 263469	Russia	Asia
ACS 28	PI 162563	China	Asia	ACS 185	PI 265513	Russia	Asia
ACS 29	PI 195121	China	Asia	ACS 186	PI 265517	Russia	Asia
ACS 32	PI 436595	China	Asia	ACS 189	PI 265522	Russia	Asia
ACS 34	PI 436598	China	Asia	ACS 191	PI 269965	Russia	Asia
ACS 36	PI 436600	China	Asia	ACS 193	PI 254698	S. America	America
ACS 38	PI 436603	China	Asia	ACS 194	PI 254698	S. America	America
ACS 40	PI 532846	China	Asia	ACS 195	PI 200113	Sri Lanka	Asia
ACS 43	PI 200108	Egypt	Africa	ACS 197	PI 253985	Syria	Asia
ACS 46	PI 298629	Egypt	Africa	ACS 204	PI 170710	Turkey	Europe
ACS 49	PI 238989	Greece	Europe	ACS 215	PI 170728	Turkey	Europe
ACS 51	PI 238991	Greece	Europe	ACS 216	PI 170729	Turkey	Europe
ACS 60	PI 156999	India	Asia	ACS 218	PI 170732	Turkey	Europe
ACS 65	PI 347847	India	Asia	ACS 220	PI 170739	Turkey	Europe
ACS 68	PI 257471	India	Asia	ACS 234	PI 175907	Turkey	Europe
ACS 70	PI 222266	Iran	Asia	ACS 241	PI 179034	Turkey	Europe
ACS 71	PI 223014	Iran	Asia	ACS 242	PI 179481	Turkey	Europe
ACS 72	PI 223411	Iran	Asia	ACS 246	PI 179490	Turkey	Europe
ACS 73	PI 227253	Iran	Asia	ACS 253	PI 238427	Turkey	Europe
ACS 74	PI 229790	Iran	Asia	ACS 278	PI 240856	Turkey	Europe
ACS 76	PI 250748	Iran	Asia	ACS 285	Landrace	Turkey	Europe
ACS 80	PI 250894	Iran	Asia	ACS 304	PI 238446	Turkey	Europe
ACS 81	PI 250944	Iran	Asia	Ozberk-82	Cultivar	Turkey	Europe
ACS 86	PI 343821	Iran	Asia	Muganli-57	Cultivar	Turkey	Europe
ACS 87	PI 343821	Iran	Asia	ACS 325	PI 254709	USA	America
ACS 88	PI 381030	Iran	Asia	ACS 326	PI 254709	USA	America

ACS 91	PI 198157	Iraq	Asia	ACS 329	PI 280791	USA	America
ACS 108	PI 285170	Israel	Asia	ACS 330	PI 280791	USA	America
ACS 109	PI 285171	Israel	Asia	ACS 331	PI 280794	USA	America
ACS 122	PI 207667	Japan	Asia	ACS 335	PI 280812	USA	America
ACS 134	PI 490031	S. Korea	Asia	ACS 337	PI 542048	USA	America
ACS 137	PI 490045	S. Korea	Asia	ACS 344	PI 599446	USA	America
ACS 139	PI 490046	S. Korea	Asia	ACS 348	PI 599455	USA	America
ACS 141	PI 490049	S. Korea	Asia	ACS 353	PI 599493	USA	America
ACS 145	PI 200107	Myanmar	Asia	ACS 356	PI 153514	Venezuela	America
ACS 152	PI 200427	Pakistan	Asia	ACS 358	PI 254702	Venezuela	America
ACS 153	PI 250228	Pakistan	Asia	ACS 359	PI 320960	Venezuela	America
ACS 155	PI 292144	Pakistan	Asia				
